# Changes in Rhizosphere Soil Fungal Communities of *Pinus tabuliformis* Plantations at Different Development Stages on the Loess Plateau

**DOI:** 10.3390/ijms23126753

**Published:** 2022-06-17

**Authors:** Jiaxing Wang, Jing Gao, Haoqiang Zhang, Ming Tang

**Affiliations:** 1 State Key Laboratory for Conservation and Utilization of Subtropical Agro-Bioresources, Guangdong Laboratory for Lingnan Modern Agriculture, Guangdong Key Laboratory for Innovative Development and Utilization of Forest Plant Germplasm, College of Forestry and Landscape Architecture, South China Agricultural University, Guangzhou 510642, China; wjx2017@nwafu.edu.cn; 2 College of Forestry, Northwest A&F University, Xianyang 712100, China; sxllgaojing@nwafu.edu.cn (J.G.); zhanghaoqiang@nwsuaf.edu.cn (H.Z.)

**Keywords:** *Piuns tabuliformis* plantation, stand age, soil properties, soil fungal community, ecological guilds, ectomycorrhizal fungus

## Abstract

The soil fungal community is an important factor in the forest ecosystems, and a better understanding of its composition and dynamic changes will contribute to the maintenance, preservation, and sustainable development of the forest ecosystems. *Pinus tabuliformis* has been widely planted for local ecological restoration on the Loess Plateau in China in recent decades. However, these plantations have been degraded to different degrees with increasing stand age. Hence, we tried to find the possible causes for the plantation degradation by analyzing soil environmental changes and soil fungal community composition at different stand ages. We collected rhizosphere soil samples from young (10-year-old), middle-aged (20-year-old), and near-mature (30-year-old) *P. tabuliformis* plantations in this region and characterized their soil properties and soil fungal community diversity and composition. Our results showed that with increasing stand age, the contents of organic carbon, ammonium nitrogen (AN) and nitrate nitrogen (NN) in the soil increased significantly, while the content of available phosphorus (AP) decreased significantly. The main factors affecting the composition of the soil fungal community were the contents of AP, AN, and NN in the soil. In addition, the genus *Suillus* was the dominant ectomycorrhizal (ECM) fungus in all periods of *P. tabuliformis* plantations in this region. The results of structural equation modeling showed that the community composition of ECM fungi was significantly correlated with stand age, soil NN, and AP contents, and that of pathogenic (PAG) fungi was significantly correlated with soil AN and AP contents. The decrease in the relative abundance of ECM fungi and the increase in the relative abundance of PAG fungi would exacerbate the degradation of *P. tabulaeformis* plantation. Our results illustrated that the content of soil AP is not only an important factor limiting the development of plantations, but it also significantly affects the community composition of soil fungi in the rhizosphere of the *P. tabuliformis* plantation. This study provides a novel insight into the degradation of *P. tabuliformis* plantations and builds a solid foundation for their subsequent management, restoration, and sustainable development on the Loess Plateau of China.

## 1. Introduction

The Chinese Loess Plateau is the largest and most concentrated loess area in the world [1]. Because of the sparse vegetation and loose soil in this area, severe soil erosion has resulted from the combined action of water and wind [2]. At the same time, the soil moisture and nutrient content of the Loess Plateau are exceedingly low, making it difficult for plants to survive there [3]. As a result, the ecological restoration of this area is extremely challenging. To curb the degradation of the ecological environment of the Loess Plateau, China has carried out a large-scale revegetation program (Grain to Green Program) since the 1990s [4]. *Pinus tabuliformis* is characterized by its strong tolerance to drought, cold, and poor soil. Since the beginning of the revegetation program, it was planted on the Loess Plateau during large-scale planting [5]. For decades, it has been considered as a pioneer tree species in forest restoration and plays a vital role in Chinese efforts to combat desertification [6]. However, with the continuous development of plantations, the *P. tabuliformis* plantations in some areas were found to be more severely degraded than those in the original areas [1]. The *P. tabuliformis* plantations in these degraded areas showed slow growth to different degrees, reduced tolerance for cold and drought, and significantly increased die-back of the trees [3]. Currently, most researchers have attributed the degradation of plantations mainly to the limitation of water resources in the soil, resulting in the formation of a dry layer of soil [1,4,7]. Some researchers have believed that the lack of nutrient elements in soil is also an important restrictive factor for the healthy development of plantations [8,9]. The factors identified in these studies have geographic and spatial constraints and cannot fully explain the degradation of plantations in this region. Therefore, an in-depth understanding of soil constraints in these degraded plantation areas is essential for plantation management and restoration.

As a bridge between above- and belowground ecosystems, soil fungi constitute an important part of terrestrial ecosystems and play an extremely crucial role in the energy flow and material cycling of ecosystems and even the entire biosphere [10,11]. Higher fungal community diversity generally means more stable ecosystems and more efficient nutrient cycling [12]. Among soil fungal communities, mycorrhizal and saprophytic (SAP) fungi are two important taxonomic groups that play key roles in nutrient cycling [13,14]. In forest ecosystems, especially for Pinaceae forests, ectomycorrhizal (ECM) fungi, as a critical fungal community, can form a mutually beneficial symbiotic relationship with host trees, promote the acquisition of nutrients and water by trees, inhibit the development of various pathogenic (PAG) fungi, and improve the resistance of trees to adversity [15,16]. Many recent studies have shown that changes in ECM fungal communities are an important factor in the development and succession of plantations [17,18,19]. SAP fungi, the main decomposers of wood, litter, and organic matter, are another important type of fungi in soil. They can release energy such as organic carbon and available nitrogen from organic substances, which is particularly important for the improvement of the soil environment [14,20]. Therefore, a systematic study of the diversity and composition (especially the functional groups) of the soil fungal community in plantation forests is needed for the long-term and effective management of plantation forest ecosystems.

The diversity and composition of soil fungi are closely related to a variety of biotic and abiotic factors [21,22]. Previous studies have shown that changes in the soil environment, such as moisture, nutrients, and pH, can significantly and directly affect the composition of soil fungal communities [23,24]. In addition, specific attention has been given to the impact of plant community succession, which alters the soil fungal composition by indirectly affecting the soil environment [25,26]. McGuire et al. [27] also indicated that soil fungal communities exhibited distinct differences in the primary forests, logged forests, and plantations. Although host specificity or preference (nonrandom association patterns) is a major driver affecting rhizosphere soil fungal composition among different plants [28], in monospecific plantations, the age of the stand may become an important determinant of the microbial community factor [17,18,19]. For example, there are significant differences in the ability to recycle and distribute different resources (C, N, and P) with changes in stand ages [29], which can significantly affect the colonization of ECM fungi and SAP fungi in the rhizosphere soil of host plants [13,30]. Nonetheless, due to the specificity and complexity between fungal species, even within the same ecosystem, different fungal ecological guilds commonly respond differently to various soil environmental conditions [31]. For these reasons, the succession of soil fungal communities is often less predictable than that of plant communities [12,32,33]. Therefore, further research is needed to understand the succession of soil fungal ecological guilds in the development stage of plantations and to determine the main influencing factors of the soil fungal community.

In this study, we investigated the soil environment and soil fungal community of *P. tabuliformis* plantations of different stand ages on the Loess Plateau and aimed to (a) understand the changing patterns of the soil environment and fungal communities in plantations affected by stand age, (b) identify the dominant ECM fungi in the rhizosphere soil of *P. tabuliformis* at different stand ages, and (c) determine the interrelationship of rhizosphere soil properties with the succession of soil fungal communities as the plantation develops.

## 2. Results

### 2.1. Soil Properties

The results of the soil properties of the rhizosphere of *P. tabuliformis* showed that significant changes in soil properties occurred with increasing stand age (Table 1). Regression analysis revealed that the AN (*p* < 0.01, R^2^ = 0.93), NN (*p* < 0.01, R^2^ = 0.67), TK (*p* < 0.05, R^2^ = 0.41), and SOC (*p* < 0.05, R^2^ = 0.53) contents in the rhizosphere soil of *P. tabuliformis* significantly increased with increasing plantation age, while the AP content (*p* < 0.05, R^2^ = 0.52) in the soil showed a decreasing trend. The AN, NN, TK, SWC, and SOC contents were highest in the PN stands, whereas AP content was highest in the PY stands. In addition, the contents of TN, TP, AK, and pH showed no virtual differences (*p* ≥ 0.05) with increasing stand age.

### 2.2. General Characterization of the Fungal Community

**Figure 1:**
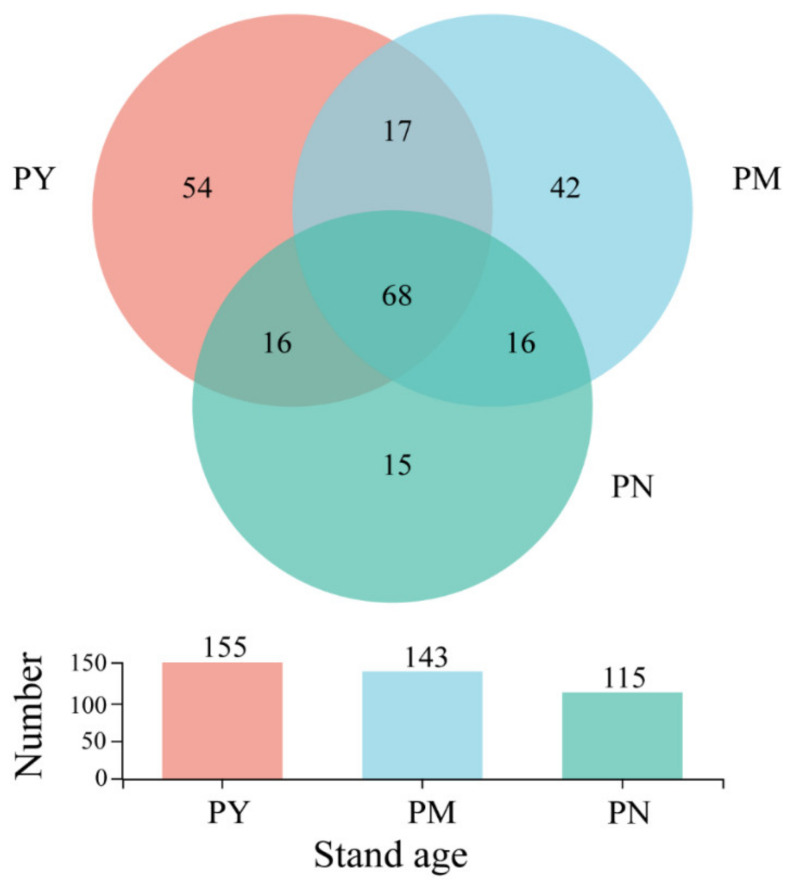
Venn diagram of the rhizosphere soil fungal composition of *P. tabuliformis* on genus level at different stand ages. PY: young forest (10a), PM: middle-aged forest (20a), and PN: near-mature forest (30a).

A total of 420,480 high-quality sequences were obtained from soil samples and were then assigned to 678 OTUs. Among them, a total of 413 fungal OTUs were identified to the genera level, which were assigned to 9 phyla, 29 classes, 74 orders, 147 families and 228 genera. Significant differences in the genera identified in the rhizosphere of *P. tabuliformis* were observed with different stand ages (Figure 1). In the rhizosphere of three different stand ages, 155, 143, and 115 fungal genera were identified. The number of fungal endemic genera identified was the highest, with 54 in the PY stands, 42 in the PM stands, and the lowest, with only 15, in the PN stands. There were 68 common genera in the rhizosphere soils of the three stand ages, accounting for 16.46% of the total.

### 2.3. Fungal Community Diversity and Structure

**Figure 2:**
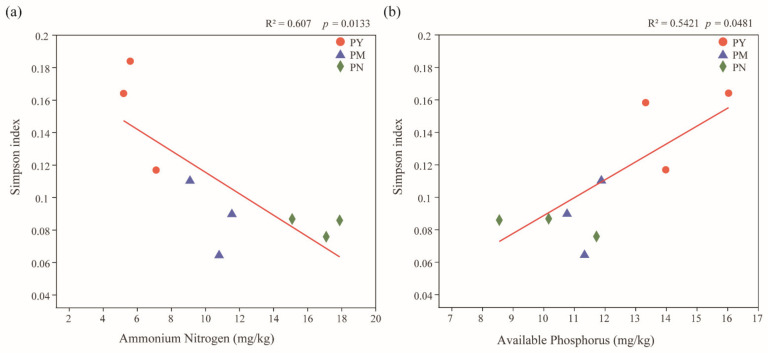
The correlation of rhizosphere soil fungal Simpson index with soil ammonium nitrogen (**a**) and available phosphorus (**b**) at different stand ages. The level of fit and the significance of the relationships are shown in the top right corner of the panels. PY: young forest (10a), PM: middle-aged forest (20a), and PN: near-mature forest (30a).

Analysis of the fungal α-diversity based on OTU data showed that stand age had a significant impact on fungal diversity (Table 2). There were significant changes (*p* < 0.001) in the diversity of fungal communities across the three stand ages, with Simpson index being highest in the PY stands compared with the other age groups. The regression analysis of the Simpson index with the soil properties showed that the Simpson index had a significant univariate linear regression relationship with the AN and AP contents (Figure 2). The Simpson index was negatively correlated with the AN content (*p* < 0.05, R^2^ = 0.61), but it was significantly positively correlated with the AP content (*p* < 0.05, R^2^ = 0.54). There was no significant correlation between other properties in the soil and the Simpson index.

**Figure 3:**
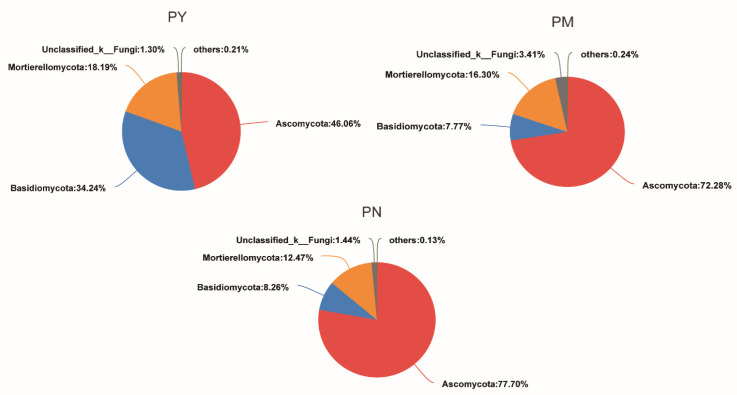
The composition of rhizosphere soil fungi of *P. tabuliformis* on phylum level at different stand ages. PY: young forest (10a), PM: middle-aged forest (20a), and PN: near-mature forest (30a).

**Figure 4:**
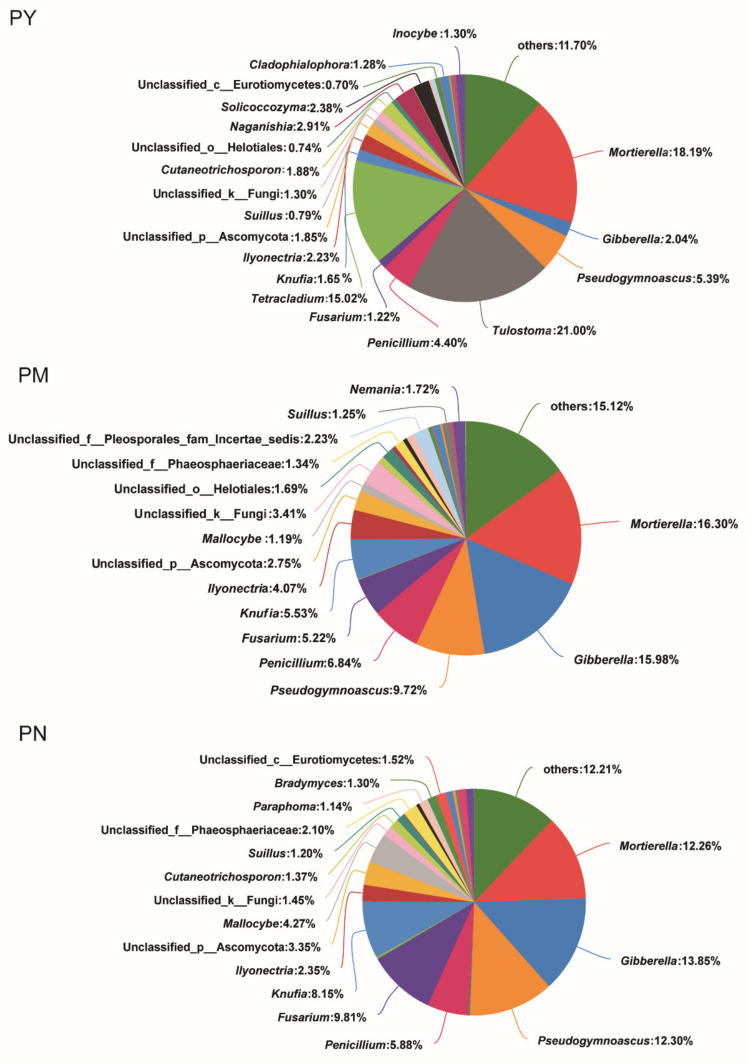
The composition of rhizosphere soil fungi of *P. tabuliformis* on genus level at different stand ages. PY: young forest (10a), PM: middle-aged forest (20a), and PN: near-mature forest (30a). The composition of rhizosphere soil fungi of *P. tabuliformis* on genus level at different stand ages.

The stand age significantly affected the composition of fungal phyla in *P. tabuliformis* rhizosphere soil (Figure 3). With increasing stand age, the abundance of Ascomycota gradually increased, the abundance of Basidiomycota significantly decreased, and the abundance of Mortierellomycota was basically the same. The relative abundance of Ascomycota was highest in the PN stands and lowest in the PY stands. The relative abundance of Basidiomycota showed the opposite trend, being highest in the PY stands and lowest in the PM and PN stands. In addition, the composition of fungal genera in the soil showed significant differences with changes in stand age (Figure 4). The dominant genera in the PY stands were *Tulostoma*, *Mortierella*, *Tetracladium*, and *Pseudogymnoascus*; the dominant genera in the PM stands were *Mortierella*, *Gibberella*, *Pseudogymnoascus*, *and Penicillium*; and the dominant genera in the PN stands were *Gibberella*, *Pseudogymnoascus*, *Mortierella*, and *Fusarium*. *Suillus* was the dominant ECM fungi genus in three stand ages, *Inocybe* was the dominant ECM fungi genus in the PY stands, and *Mallocybe* was the dominant ECM fungi genera in the PM and PN stands. The relative abundance of *Suillus* in the PM and PN stands was higher than that in the PY stands, and the relative abundance of *Mallocybe* was highest in the PN stands.

**Figure 5:**
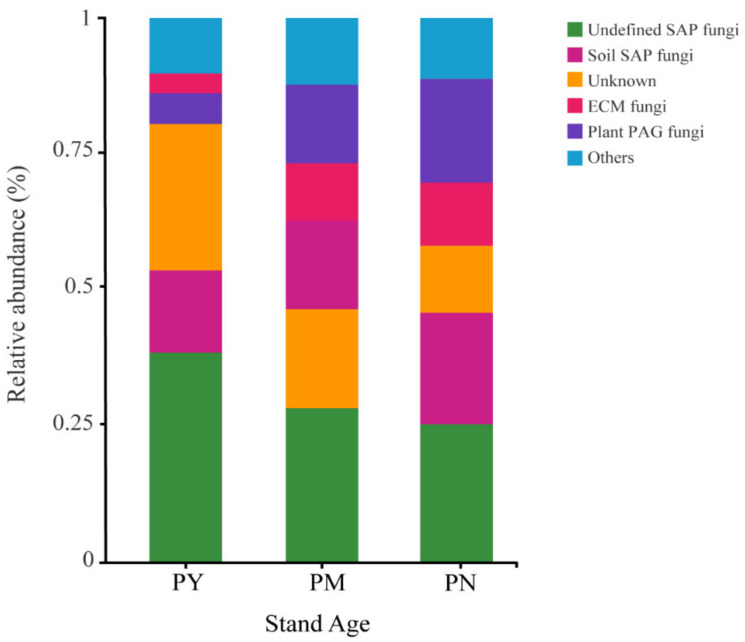
Functional taxonomic composition of rhizosphere soil fungal communities of *P. tabuliformis* at different stand ages. SAP: saprotrophic, ECM: ectomycorrhizal, PAG: pathogenic, PY: young forest (10a), PM: middle-aged forest (20a), and PN: near-mature forest (30a).

After classifying and analyzing the functions of fungi based on the OTU results, we found that the functional composition of fungi changed significantly with different stand ages (Figure 5). In the PY stands, the relative abundance of fungi that have not been determined to be functionally classified was significantly higher than that in the PM and PN stands. With increasing stand age, the relative abundance of SAP fungi, plant PAG fungi, and ECM fungi increased significantly. The relative abundance of plant PAG fungi was the highest in the PN stands, and the relative abundance of SAP fungi and ECM fungi was not significantly different in the PM and PN stands.

**Figure 6:**
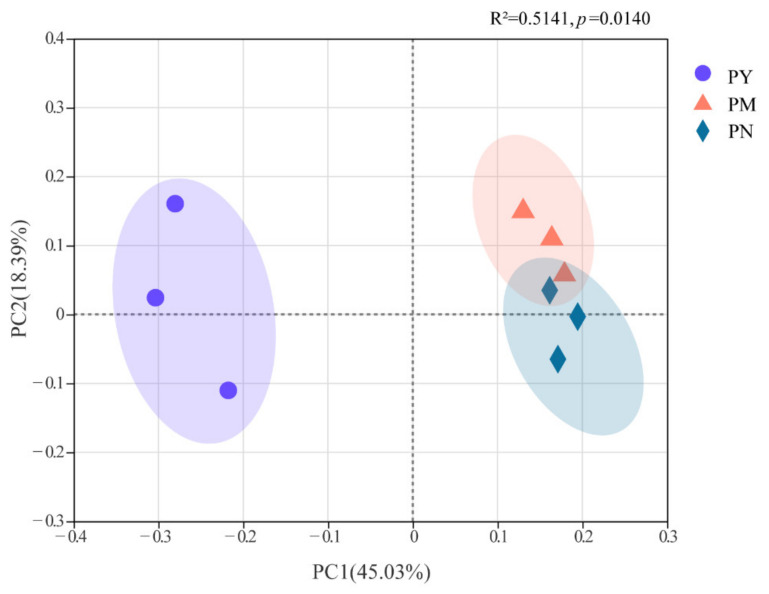
Principal coordinates analysis of the composition of rhizosphere soil fungal community of *P. tabuliformis* at different stand ages. The level of fit and the significance of the relationships are shown in the top right corner of the panels. PY: young forest (10a), PM: middle-aged forest (20a), and PN: near-mature forest (30a).

**Figure 7:**
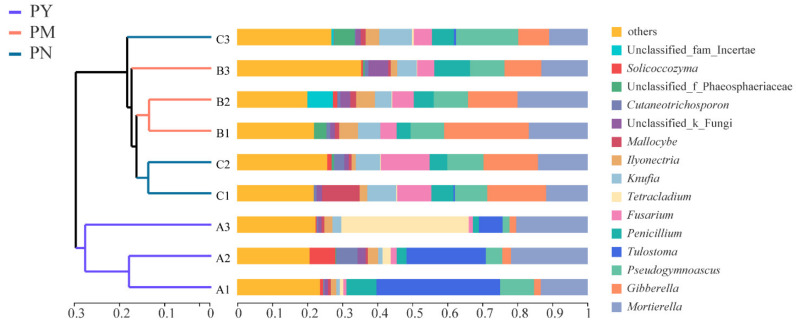
Hierarchical clustering analysis of the composition of rhizosphere soil fungal community of *P. tabuliformis* at different stand ages. PY: young forest (10a), PM: middle-aged forest (20a), and PN: near-mature forest (30a).

The results of PCoA showed that there were significant differences in the fungal community composition among different ages (R^2^ = 0.51, *p* < 0.05), and stand age could significantly affect the rhizosphere soil fungal community structure (Figure 6). The soil fungal community structure in the PY stands was clearly separated from that of PM and PN, while the soil fungal communities of PM and PN were relatively close. The results from the hierarchical clustering analysis also confirmed that the soil fungal community structure in the PY stands was significantly different from that in the PM and PN stands, while the soil fungal community structures of PM and PN were relatively similar, in which the genera *Tetracladium*, *Fusarium* and *Knufia* were the main factors for this difference (Figure 7).

### 2.4. Associations between Fungal Community and Soil Properties

**Figure 8:**
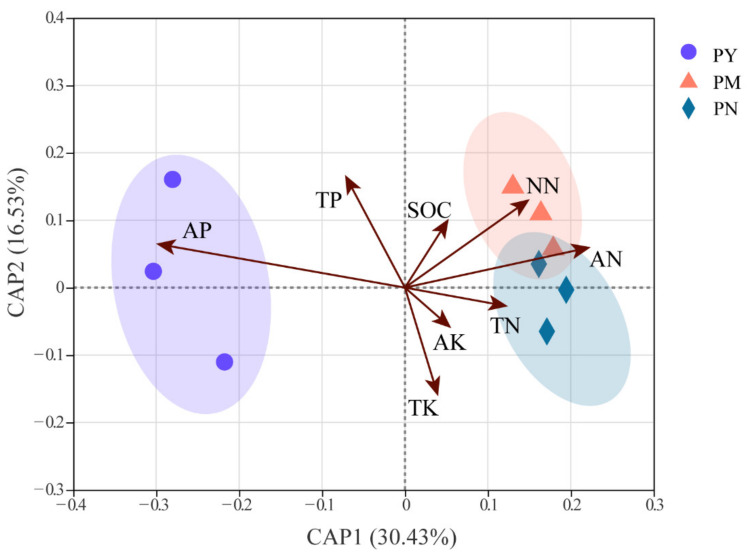
Distance-based redundancy analysis of the composition of rhizosphere soil fungal community of *P. tabuliformis* at different stand ages. TN: total nitrogen, NN: nitrate nitrogen, AN: ammonium nitrogen, TP: total phosphorus, AP: available phosphorus, TK: total potassium, AK: available potassium, SOC: soil organic carbon, PY: young forest (10a), PM: middle-aged forest (20a), and PN: near-mature forest (30a).

The results of the db-RDA showed the relationship between fungal community composition and soil properties in the rhizosphere of *P. tabuliformis* at different stand ages (Figure 8). More than 46.96% of the variance in the fungal community could be explained according to the two canonical axes CAP 1 and CAP 2. CAP 1 and CAP 2 accounted for 30.43% and 16.53% of the variation, respectively. According to the db-RDA diagram, the significant soil properties affecting the soil fungal community were the AP (*p* < 0.01), AN (*p* < 0.05), and NN (*p* < 0.05) contents. In addition, the fungal communities in the PY stand were significantly and positively correlated with the soil AP contents, whereas the communities in the PM and PN stands were significantly positively correlated with the soil AN and NN contents. These observations suggested that soil AN, NN and AP contents were the major factors associated with variation in rhizosphere fungal community composition.

**Figure 9:**
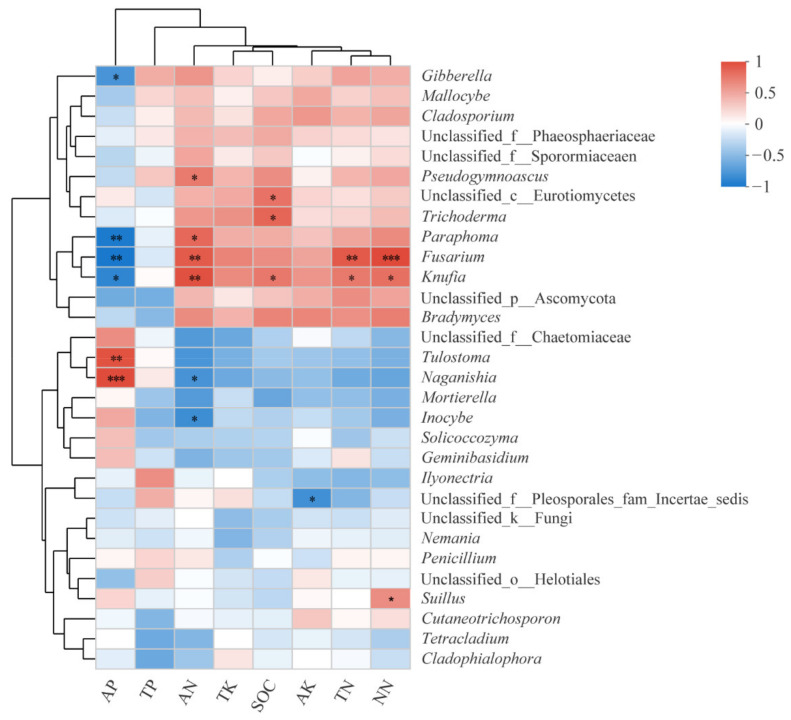
Correlation analysis between soil fungal community composition and soil properties in the rhizosphere of *P. tabuliformis*. AP: available phosphorus, TP: total phosphorus, AN: ammonium nitrogen, TK: total potassium, SOC: soil organic carbon, AK: available potassium, TN: total nitrogen, NN: nitrate nitrogen, *: significant at *p* < 0.05, **: significant at *p* < 0.01, and ***: significant at *p* < 0.001.

The results of the correlation analysis between the rhizosphere soil fungal community and soil properties showed that the relative abundance of different soil fungal genera was significantly related to different soil properties (Figure 9). Among them, the abundances of *Pseudogymnoascus*, *Paraphoma*, *Fusarium*, and *Knufia* were significantly positively correlated with the AN content, and the abundances of *Fusarium*, *Knufia*, and *Suillus* were significantly positively correlated with the NN content. In contrast, the abundances of *Gibberella*, *Paraphoma*, *Fusarium*, and *Knufia* were significantly negatively correlated with the AP content, and the abundances of *Naganishia* and *Inocybe* were significantly negatively correlated with the AN content.

**Figure 10:**
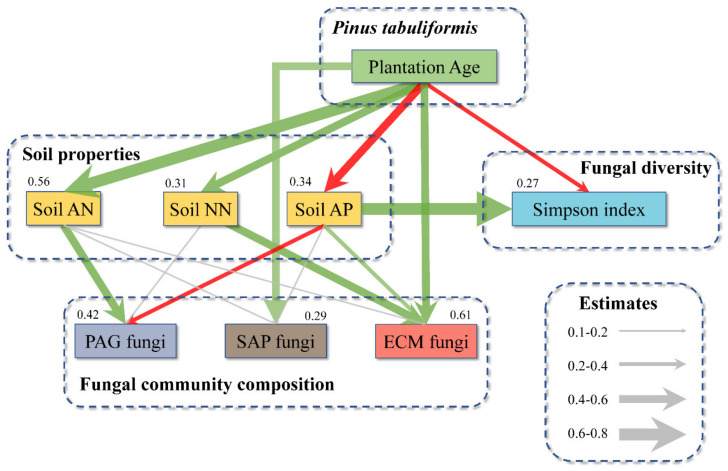
Structural equation model of the relationships among stand ages, soil properties, fungal community composition and fungal diversity (*p* = 0.425, Goodness-of-fit index = 0.726, *n* = 27). The number on each component in the model is R^2^ (the proportion of variance explained), the thickness of the arrow indicates the size of path coefficients, the green and red arrows indicate positive and negative correlations, respectively, and the gray arrow indicates no significant correlation. AN: ammonium nitrogen, AP: available phosphorus, NN: nitrate nitrogen, PAG: pathogenic, SAP: saprotrophic, and ECM: ectomycorrhizal.

SEM provided good fits for the fungal community composition and diversity data, as indicated by the *p* and GFI (Goodness-of-fit index) metrics (Figure 10). The SEM results indicated that the increase in stand age significantly increased the soil AN and NN contents and the relative abundance of SAP fungi and ECM fungi but reduced the soil AP content and fungal diversity. In addition, the increase in soil AN increased the abundance of PAG fungi. The increase in soil NN content increased the abundance of ECM fungi. In addition, the fungal diversity and abundance of ECM fungi were also positively affected by the soil AP content, while the abundance of PAG fungi was negatively affected. These results indicated that the stand age of *P. tabuliformis* could directly and indirectly influence the soil fungal community (composition and diversity) by affecting soil properties.

## 3. Discussion

Soil is the basis for plant growth and development, and plant growth has an important influence on soil physicochemical properties and soil biological processes [11]. Previous studies have shown that afforestation significantly affects the properties of plant rhizosphere soils, and this effect varies with stand age [9,17,18,34]. In this study, the AN, NN, TK and SOC contents in the rhizosphere soil of *P. tabuliformis* increased significantly with increasing stand age. The establishment of a plantation ecosystem can enable the development of high net primary productivity and reduce the degree of soil disturbance, which leads to increased C, N, and P storage in soil [35,36]. This process is also largely influenced by the yield, quality, and rate of decomposition of plantation litter. With increasing stand age (20-30a), the biomass of the *P. tabuliformis* forest increased, and its understory litter increased significantly. At this time, the microorganisms and root exudates in the soil can continuously accumulate, decompose the litter in the ground soil, and gradually release the organic nutrients in the litter, which leads to a continuous increase in the AN, NN, and SOC contents in the soil [24]. The accumulation of organic carbon and nitrogen in the soil indicates that the nutritional status of the soil improves with the increasing age of *P. tabuliformis*, suggesting that stand age plays an important role in the restoration of the Loess Plateau ecosystem [37]. However, in our study, with the increase in stand age, although nutrient status in terms of organic carbon and available nitrogen of the rhizosphere soil of *P. tabuliformis* improved, the content of AP in the soil decreased significantly. This is also consistent with previous studies, in which the depletion of AP in the soil by trees increases further with the age of the forest. The soil phosphorus content was shown to be an important limiting factor in the process of soil ecological restoration on the Loess Plateau [35,37,38]. The lack of phosphorus replenishment by weathering of mineral rocks leads to a significant reduction in AP in the soil [39]. Therefore, we believe that improving the content of AP in the soil of the *P. tabuliformis* plantation on the Loess Plateau to meet the needs of subsequent high forest productivity will be a priority for ecosystem management in this area.

The composition and structure of soil fungal communities are influenced by the interaction of multiple biotic and abiotic factors [21,22], including changes in forest stand age [40,41,42,43]. In this study, although the rhizosphere soil fungal richness and Shannon index of *P. tabuliformis* did not change significantly, the Simpson index decreased significantly with increasing stand age. Notably, the lowest Simpson index of root-associated fungi was observed in the PN stands. These results are consistent with the findings of Koizumi et al. [44] and Dong et al. [17]. In the early stage of plantation establishment, there are many early fungi in the rhizosphere soil of trees [9]. With the succession of plantations and continuous changes in the soil environment, some fungi gradually occupy a dominant position, inhibiting the reproduction of other fungi [31]. Therefore, fungi that exist in the later stages of forest succession tend to be more adaptable to changes in the soil environment [45]. In addition, previous studies have demonstrated that the extractable P and N concentrations in the soil are important predictors of fungal diversity [46,47]. This hypothesis is further supported by our findings, which showed that the AN and AP contents in the soil were significantly correlated with the Simpson’s index of the fungal community. In addition, the results of this study showed that in the nearby mature *P. tabuliformis* plantation on the Loess Plateau, the effect of P content in soil on fungal diversity was greater than that of N.

The community composition of rhizosphere fungi differed among the different age groups, especially those from PY and others stands, as indicated by the PCoA ordination. These results are consistent with previous studies by Dong et al. [17] and Guo et al. [18] on the rhizosphere fungal composition in different stand ages of *Pinus massoniana* and *Pinus sylvestris*, respectively. Ascomycota, the most diverse phylum in the fungi kingdom [48], is the dominant phylum in many soil fungal communities [9,18,49]. In our study, both Ascomycota and Basidiomycota were dominant phyla in the early pine plantations, but with increasing forest age, the relative abundance of Basidiomycota decreased significantly, and Ascomycota gradually took the dominant position. Nevertheless, the fungal composition of the rhizosphere of *P. tabuliformis* still showed great heterogeneity and volatility at the genus level. These results suggest that the succession of plant rhizosphere fungal communities is complex and unpredictable not to mention affected by a variety of other factors [42].

Through the analysis of fungal functional groups, we found that the functional composition of fungi changed significantly with increasing stand age, especially the composition of ECM fungi and PAG fungi. The relative abundance of ECM fungi, SAP fungi, and PAG fungi was higher in the PM and PN stands, whereas the relative abundance of fungi with no defined function was lower in the PM and PN stands. Previous studies have suggested that ECM fungi and SAP fungi generally occupy relatively high abundances in more mature forests because they play an important role in the energy flow of material cycles in forests [17,18,19,24]. Plantation development and soil fungal community dynamics have close interactions and cooperative evolution [9,42]. With the gradual maturity of the plantation, the accumulation of a large amount of organic matter under the tree is more conducive to the reproduction of these two types of fungi than the early stage of the plantation [45].

In our study, *Suillus* was the dominant ECM fungal genus in all stands, *Inocybe* was the dominant ECM fungal genus in the PY stands, and *Mallocybe* was the dominant ECM fungal genus in the PM and PN stands. As in previous findings, *Suillus*, the most prevalent ECM fungal genus associated with pine [50], is essential for the growth of *P. tabuliformis* on the Loess Plateau at all stages. *Inocybe* can only coexist with *P. tabuliformis* in the PY stands and exists abundantly in the soil. With increasing stand age, *Inocybe* was gradually replaced by *Mallocybe*. A similar ECM fungal taxa replacement across a primary successional gradient was reported by Davey et al. [51], where early successional species such as *Laccaria* spp. and *Hebeloma* spp. (Agaricales) were replaced by *Tomentella* spp. (Thelephorales). We can speculate that *Inocybe* colonized trees at the youngest sites in our chronosequence, which was later replaced by *Mallocybe*. The crucial ECM fungi in the maturation stage of *P. tabuliformis* plantations on the Loess Plateau were *Suillus* and *Mallocybe*.

In contrast to the changes in ECM fungi, the dominant SAP genera changed continuously with increasing stand age. These results suggest that functional fungal groups (e.g., ECM and SAP fungi) in plant rhizosphere soils respond differently to stand development, and certain dominant genera may vary with stand age [17,27]. It is worth noting that the abundance of PAG fungi (e.g., *Gibberella* and *Fusarium*) in the soil increased significantly with stand age. *Gibberella* is a well-known pathogen that has been implicated in the diseases of several plants [52]. Although some species of *Fusarium* are saprotrophic in soil, almost all species of *Fusarium* were found to be pathogenic to plants in our study, especially *Fusarium proliferatum* and *Fusarium solani*, which are the main causes of plant root rot [53]. Based on these results, we speculate that the decline in *P. tabuliformis* plantations on the Loess Plateau may also be exacerbated by the significant increase in these PAG fungi in the soil.

The planting of *P. tabulaeformis* plantations on the Loess Plateau has changed soil properties greatly [9], which has a substantial impact on soil fungal communities in forest [18,24,43]. According to our db-RDA results, the soil NN, AN and AP were indicated as the main factors influencing the composition of the fungal community. These results are partially in line with several studies describing a strong link between soil properties and the species richness and community composition of soil fungi [17,23,24,54]. Although these soil properties are important influencing factors for fungal community composition, fungi have different responses to different soil physicochemical properties due to their different physiological characteristics [55,56]. In our study, ECM fungi, especially *Suillus*, were found to be significantly positively correlated with soil NN, which proved that soil N was the main factor affecting the ECM fungal community [54], and that the increase in NN content contributed to the colonization of ECM fungi in the poor soil environment [57]. ECM fungi clearly have the potential to contribute significantly to P mobilization from sparingly soluble inorganic and organic sources in soil [58]. Previous studies have shown that high P concentrations reduce the abundance of ECM fungi in soil [59]. However, Clausing et al. [60] indicated that the abundance of ECM fungi in low-P soils increased with increasing P concentrations. Our findings agree with the conclusion that P, as the main soil nutrient-limiting factor, also limits the proliferation of ECM fungi in the soil with increasing stand age in *P. tabuliformis* plantation on the Loess Plateau. At the same time, we also found that there was a significantly negative correlation between the AP content in the soil and the abundance of PAG fungi. Therefore, we speculate that increasing the availability of P in the soil in this area can not only promote the growth of plants and increase the abundance of ECM fungi but can also decrease the abundance of PAG fungi. These details are essential for strengthening our management of *P. tabuliformis* plantations and are critical for potential policy making aimed at restoring the ecological environment of the Loess Plateau in China.

## 4. Materials and Methods

### 4.1. Study Site

The study site was located in Fugu County (39°20′ N, 110°28′ E) in Shaanxi Province, northwestern China, which borders the Inner Mongolia Plateau on the northeastern Loess Plateau. The region belongs has a warm temperate semiarid continental monsoon climate. The average annual precipitation is 453.5 mm, mainly from July to September, accounting for 67% of the annual precipitation. At the same time, its evaporation is 1500–2000 mm, which is significantly higher than that of precipitation. The average annual temperature is 9.1 °C, and the frost-free period averages 177 days. The study area is usually covered by fixed and semifixed loess and sand, and the dominant soil textures are sand and loamy sand. Since the large-scale revegetation program began in the 1990s, *P. tabuliformis* pure plantations have been planted on a large scale in this region, which has been effectively protected by measures such as grazing prohibition, logging prohibition, and fire prevention, resulting in the formation of numerous *P. tabuliformis* plantations of different stand ages. Nevertheless, with the continuous development of plantations, the *P. tabuliformis* plantations experienced varying degrees of degradation in this area.

We adopted the method of time–space substitution to establish a new chronological sequence of *P. tabuliformis* plantations for experiments. We conducted a detailed survey of the stands in this area and measured various data in the plantations, such as elevation, slope positions and aspect, soil type, etc., to ensure that the selected sample stands have similar ecological conditions. After professional evaluation, we selected *P. tabuliformis* pure plantations at 3 different developmental stages (10, 20, and 30 years old) for research, which are referred to as young forest (PY), middle-aged forest (PM) and near-mature forest (PN), respectively. All the *P. tabuliformis* plantations had similar conditions, and three plots (400 m × 400 m) were set for each stand age, which are separated from each other by more than 200 m. The canopy density in the plantations was 0.5–0.6. The basic conditions of the sample sites are summarized in Table 3. There were a few herbaceous plants in the study area with the dominant species being *Artemisia scoparia* Waldst *et* Kit., *Digitaria sanguinalis* (L.) Scop., *Setaria viridis* (L.) P. Beauv, and *Eleusine indica* (L.) Gaertn [3].

### 4.2. Collection and Analysis of Soil Samples

At each sample plot, we set up five 20 m × 20 m quadrats and adopted the five-point sampling method for sampling in each quadrature to ensure the independence of the pine samples. The litter and debris on the ground around the trunk were removed, and four soil cores (20 cm wide, 50 cm deep) were selected in four different directions for sampling. The fine roots directly connected to the main root of *P. tabuliformis* in the soil core were collected, and the soil attached to the surface of the fine roots was carefully collected with clean tweezers as a rhizosphere soil sample. Then, the four repeated soil samples in the same tree were mixed to create one mixed soil sample (approximately 200 g). The 25 different mixed soil samples were then combined into a composite sample. Three composite samples were used for subsequent analysis at each stand ages. All samples were placed in sealed bags and kept frozen at −20 °C until processing. In a subsequent analysis, part of the soil sample was used to analyze soil properties, and the other part was used to analyze the soil fungal community composition.

To determine whether variation in soil properties was associated with changes in the stand age of the *P. tabuliformis* plantation, we measured a series of soil properties of each composite sample including the total nitrogen (TN), ammonium nitrogen (AN), nitrate nitrogen (NN), total phosphorus (TP), available phosphorus (AP), total potassium (TK), available potassium (AK), soil organic carbon (SOC) contents, soil water content (SWC), and pH. The soil samples were air dried at 20–25 °C, some of which were passed through a 1 mm sieve, while others were passed through a 0.25 mm sieve. The TN content was measured according to the Kjeldahl digestion procedure [61]. After leaching with a potassium chloride solution, the contents of NN and AN were determined according to a colorimetric method and analyzed by automatic flow injection [62]. The TP content was measured using molybdenum antimony blue colorimetry [63]. The AP was extracted with sodium bicarbonate and analyzed via a spectrophotometer [64]. The TK content was digested with hydrofluoric acid and perchloric acid, and AK was extracted with 1 N ammonium acetate and analyzed by atomic absorption spectroscopy. The SOC content was measured using dichromate oxidation [65]. The SWC was measured using the 105 °C drying method [66]. The soil pH was measured in distilled water using a 1:2.5 soil/solution ratio [67].

### 4.3. Sampling and Molecular Characterization of Fungi

Fungi occurring in the soils were sampled from the composite soil samples. Total DNA was extracted from each soil sample by using an E.Z.N.A.^®^ Soil DNA Kit (Omega Bio-Tek, Norcross, GA, USA). The DNA extract was tested on a 1% agarose gel. The concentration and purity of the DNA were assessed using a NanoDrop 2000 UV-vis spectrophotometer (Thermo Scientific, Wilmington, DE, USA). The internal transcribed spacer (ITS) region of nuclear rDNA was amplified with the primer pairs ITS1F (5′-CTTGGTCATTTAGAGGAAGTAA-3′) and ITS2R (5′-GCTGCGTTCTTCATCGATGC-3′) by an ABI GeneAmp^®^ 9700 PCR thermocycler (Applied Biosystems, Foster City, CA, USA). PCR amplification was carried out as follows: initial denaturation at 95 °C for 3 min, followed by 30 cycles of denaturing at 95 °C for 30 s, annealing at 55 °C for 30 s and extension at 72 °C for 45 s and a single extension at 72 °C for 10 min, with a final stop at 4 °C. The volume for the PCR was 20 μL, containing 6.6 μL of PCR Mix (Transgen Bio-Technology Company, Beijing, China), template DNA (10 ng), 0.8 μL of each primer (5 μM; ITS1F and ITS2R), and a proper amount of ddH_2_O (fill up to 20 μL in total). PCR was carried out in triplicate. The PCR products were separated by 2% agarose gel electrophoresis, purified with the AxyPrep DNA Gel Extraction Kit (Axygen Biosciences, Union City, CA, USA) according to the manufacturer’s instructions and quantified using a Quantus™ Fluorometer (Promega, Madison, WI, USA). Purified amplicons were pooled in equimolar amounts and paired-end sequenced on an Illumina MiSeq PE300 platform (Illumina Corporation, San Diego, CA, USA) according to the standard protocols by Majorbio Bio-Pharm Technology Co., Ltd. (Shanghai, China).

### 4.4. Data Preprocessing and Bioinformatics Analysis

The raw reads were demultiplexed, quality filtered by fastp Version 0.20.0, and merged by FLASH Version 1.2.7. The sequences were categorized into operational taxonomic units (OTUs) defined by 97% similarity using UPARSE Version 7.1 (http://drive5.com/uparse/, accessed on 18 January 2022) after screening, filtering, preclustering, and chimera removal. To extract taxonomic information, the RDP Classifier (Version 2.2, http://sourceforge.net/projects/rdp-classifier/, accessed on 23 January 2022) algorithm was applied to each OTU’s representative sequence.

The FUNGuild database (https://github.com/UMNFuN/FUNGuild/, accessed on 5 February 2022) was used to annotate fungal functions [68]. The FUNGuild software annotates taxonomic data in the OTU table with relevant data in its online database, including guild, trophic mode, and growth morphology [69]. We only used data with confidence scores of “Probable” and “Highly Probable”. The functionality prediction was made using data from existing studies.

### 4.5. Statistical Analysis

The statistical significance of the differences in soil properties and soil fungal diversity and composition among stand ages was determined by one-way analysis of variance (ANOVA) using R software (V.4.0.4). Tukey’s multiple range tests were performed to assess statistically significant differences between values at a 95% confidence level. Alpha-diversity (α-diversity) was used to examine the complexity of species diversity in the sample using three indices (Sobs, Simpson, and Shannon). The Sobs index is the number of detected OTUs used to analyze fungal community richness, and the Simpson and Shannon indices were used to analyze fungal community diversity. The interactions between the Simpson index and soil properties were analyzed by regression analysis using R software (V.4.0.4). Principal coordinate analysis (PCoA) was applied to visualize the fungal community structure in relation to each stand age using R software (V.4.0.4, Vegan package). Distance-based redundancy analysis (db-RDA) was used to investigate the importance of environmental factors in explaining the distribution patterns of fungal communities for different stand ages using R software (V.4.0.4, Vegan package). Heatmap correlation analysis was performed to evaluate the relationships between soil properties and the soil fungal community using R software (V.4.0.4, Pheatmap package). Structural equation modeling (SEM) was applied to clarify the associations of stand age with soil fungal community composition, diversity and soil properties. The model was established based on our current knowledge of the impact of environmental variables on soil microbial communities and was appropriately revised in the subsequent analysis process. The SEM analyses were performed using AMOS Version 20.0 (Amos Development Corporation, Meadville, PA, USA).

## 5. Conclusions

On the Loess Plateau, the soil nutrient status, especially the carbon and nitrogen contents, significantly improved with increasing stand age in the *P. tabuliformis* plantation. However, the content of AP in the soil decreased and became the main limiting factor for plant growth in this area. Meanwhile, the composition and diversity of the fungal community in the rhizosphere soil of *P. tabuliformis* were significantly different with increasing stand age. The ECM fungus *Suillus* was the dominant fungus in the rhizosphere soil of *P. tabuliformis* at all stand ages. The functional composition of soil fungi had different sensitivities to soil properties, and AP, as an important factor, significantly affected the relative abundance of ECM fungi and PAG fungi. However, the current experimental data are limited to 10- to 30-year-old *P. tabuliformis* plantations. Further continuous research is still needed in the future development of Loess Plateau plantations. Overall, our results revealed the relationship between taxonomic fungal composition and soil properties during plantation development stages, which provides insights into recommendations for future plantation management and ecological restoration in this area.

## Figures and Tables

**Table 1:** The rhizosphere soil properties of *P. tabuliformis* at different stand ages.

Sites	TN (mg/kg)	AN (mg/kg)	NN (mg/kg)	TP (mg/kg)	AP (mg/kg)	TK (g/kg)	AK (mg/kg)	SOC (g/kg)	SWC (%)	pH
PY	173.10 ± 11.97a	5.97 ± 0.82c	1.88 ± 0.07b	157.43 ± 14.27a	13.97 ±1.71a	13.34 ± 1.25b	43.76 ± 5.67a	5.66 ± 0.51b	4.23 ± 0.47b	8.31 ± 0.07a
PM	179.97 ± 23.40a	10.50 ± 1.03b	2.01 ± 0.09b	174.49 ±11.77a	11.34 ± 0.45b	13.59 ± 1.61b	40.80 ± 8.12a	4.66 ± 0.44b	3.85 ± 0.96b	8.38 ± 0.03a
PN	245.43 ± 33.03a	16.71 ± 1.17a	2.87 ± 0.31a	160.54 ± 9.72a	10.16 ±1.29c	17.31 ± 1.49a	54.60 ± 2.97a	12.18 ± 1.48a	6.38 ± 0.86a	8.34 ± 0.05a
*p* value	ns	Linear regression (*p* < 0.01, R^2^ = 0.93)	Linear regression (*p* < 0.01, R^2^ = 0.67)	ns	Linear regression (*p* < 0.05, R^2^ = 0.52)	Linear regression (*p* < 0.05, R^2^ = 0.41)	ns	Linear regression (*p* < 0.05, R^2^ = 0.53)	ns	ns

Note: Data expressed as mean ± standard deviation (*n* = 3). PY: young forest (10a), PM: middle-aged forest (20a), and PN: near-mature forest (30a). Different lowercase letters indicate significant differences with a *p* value < 0.05 based on the analysis of variance, “ns” indicates the regression analysis results were not significant (*p* ≥ 0.05).

**Table 2:** The rhizosphere soil fungal diversity index of *P. tabuliformis* at different stand ages.

Sites	Sobs Index	Shannon Index	Simpson Index
PY	94.7 ± 22.1a	2.7 ± 0.1a	0.14 ± 0.03a
PM	81.7 ± 12.8a	3.1 ± 0.2a	0.06 ± 0.02b
PN	72.3 ± 9.5a	3.0 ± 0.2a	0.06 ± 0.01b
*p* value	0.562	0.704	*p* < 0.001

Note: Data expressed as mean ± standard deviation (*n* = 3). PY: young forest (10a), PM: middle-aged forest (20a), and PN: near-mature forest (30a). Different lowercase letters indicate significant differences with a *p* value < 0.05 based on the analysis of variance.

**Table 3:** Description of the sampling sites.

Sites	Stand Age	Height (m)	Diameter at Breast Height (cm)	Canopy Density	Planting Density (N/ha)
PY	10a	4.32 ± 0.71	10.54 ± 0.31	0.46 ± 0.05	954 ± 52
PM	20a	9.15 ± 0.47	12.36 ± 0.60	0.53 ± 0.09	1003 ± 63
PN	30a	12.33 ± 0.26	14.73 ± 0.85	0.63 ± 0.08	808 ± 86

Note: PY: young forest (10a), PM: middle-aged forest (20a), and PN: near-mature forest (30a). Data expressed as mean ± standard deviation (*n* = 3).

## Data Availability

Not applicable.
